# Drought Dominates over Warming in Structuring Growth and Defense in Invasive *Solidago canadensis* vs. Native *Solidago decurrens*

**DOI:** 10.3390/plants15182746

**Published:** 2026-09-08

**Authors:** Jeffrey Okundi, Ling Yuan, Guanlin Li, Lyuben Zagorchev, Daolin Du, Junmin Li

**Affiliations:** 1 School of Environment and Safety Engineering, Jiangsu University, Zhenjiang 212013, China; okundi2003@yahoo.com (J.O.); guanlinli1207@163.com (G.L.); 2 Zhejiang Key Laboratory for Restoration of Damaged Coastal Ecosystems, School of Life Sciences, Taizhou University, Taizhou 318000, China; lingyuan@tzc.edu.cn; 3 Department of Biochemistry, Faculty of Biology, Sofia University “St. Kliment Ohridski”, 8 Dragan Tsankov blvd., 1164 Sofia, Bulgaria; lzagortchev@yahoo.co.uk; 4 Department of Life Sciences and Health, Huzhou College, Huzhou 313000, China

**Keywords:** carbon allocation, drought, herbivory, root-to-shoot ratio, structural carbohydrates, piecewise structural equation model, *Solidago canadensis*

## Abstract

Plants are increasingly exposed to simultaneous warming, drought, and herbivory, yet how invasive and native species regulate carbon allocation under these interacting stressors remains poorly understood. We conducted a full-factorial greenhouse experiment manipulating temperature (+3 °C), soil water availability, and herbivory to compare carbon allocation responses in the invasive *Solidago canadensis* and its native congener *S. decurrens*. Drought emerged as the dominant driver of carbon reallocation, shifting investment away from biomass production and photosynthetic function toward belowground allocation, osmotic adjustment, defense, and structural reinforcement. Herbivory imposed additional carbon demands and modified drought-induced responses, particularly by constraining phenolic accumulation under combined stress, whereas warming had comparatively weaker and largely context-dependent effects. Our results showed that the two species differed in how carbon was distributed under stress: *S. canadensis* maintained greater biomass, chlorophyll, soluble sugars, and hemicellulose, whereas *S. decurrens* showed stronger investment in structural components. Structural equation models further revealed contrasting pathways linking physiological, defensive, and structural traits to biomass, supporting species-specific strategies of carbon allocation under multiple stressors. These findings suggest that the greater carbon-allocation flexibility observed in *S. canadensis* may contribute to maintaining performance under increasingly variable environmental conditions. However, because *S. canadensis* was raised directly from field-collected seeds without a refresher generation, potential maternal environmental effects cannot be completely excluded. Overall, our findings identify coordinated redistribution of carbon among competing functional pools as a potential mechanism underlying contrasting stress responses in these two congeneric species.

## 1. Introduction

Plants frequently encounter simultaneous biotic and abiotic pressures, such as temperature, water availability, and herbivory. These factors rarely operate independently, and their combined effects on plant growth and metabolism can be additive, antagonistic, or synergistic, making responses to multiple stressors difficult to predict from single-factor studies [1]. Biological invasions represent an important component of global change, with invasive species altering ecosystem processes, species interactions, and resource use patterns across environmental gradients [2,3]. Although invasive plants frequently differ from native species in growth, resource use, and stress-response strategies [4,5], evidence for a consistent invasive advantage under environmental stress remains mixed and depends on the identity, intensity, and combination of stressors. Understanding invasion under ongoing global change therefore requires moving beyond comparisons of individual stress responses to determine how invasive and native plants coordinate growth, resource allocation, and stress tolerance when multiple environmental pressures occur simultaneously.

Plant responses to environmental stress are governed by the allocation of carbon among competing sinks, including growth, structural reinforcement, chemical defense, and metabolic maintenance. This carbon allocation framework provides a mechanistic basis for understanding plant responses to environmental constraints [6,7]. Allocation theories predict that plants shift carbon toward functions that enhance acquisition of limiting resources or improve survival under stress [8,9]. Empirical studies show that these allocation patterns can differ between invasive and native species. For example, invasive populations of *Triadica sebifera* exhibited reduced allocation to structural carbohydrates but increased allocation to soluble carbon compared to native populations, indicating greater flexibility in carbon use under stress [10]. Similarly, invasive species such as *Ailanthus altissima* have been shown to maintain higher soluble sugar concentrations under drought, supporting osmotic regulation and continued growth [11]. In contrast, native species often exhibit stronger shifts toward conservative allocation strategies, including increased investment in structural components or belowground biomass under resource limitation [12]. Consistent with this pattern, comparative studies have found invasive plants positioned toward more resource-efficient strategies, whereas native species may favor safer, more conservative resource-use strategies under water limitation [13,14]. However, other studies show substantial species-specific variation in these responses, indicating that neither greater mobile-carbon investment nor conservative allocation represents a universal invasive or native strategy [15,16]. These contrasting patterns suggest that differences in carbon allocation strategies may underlie variation in stress tolerance and performance between invasive and native plants. This also emphasizes that such differences are context-dependent rather than universally associated with invasion status.

Structural carbon allocation represents a major component of plant response to environmental stress. Cell-wall constituents, including hemicellulose, cellulose, and lignin, account for substantial carbon investment and determine tissue strength and function [17,18]. Drought commonly induces cell-wall remodeling, including increased lignification or altered polysaccharide composition, which can enhance structural stability and resistance to hydraulic stress [19,20]. However, structural responses are not uniform across species and are not consistently stronger in invasive plants. For example, invasive *Triadica sebifera* populations showed reduced cellulose but increased soluble carbon compared to native populations, suggesting a shift toward more flexible carbon use [10]. Conversely, comparative evidence indicates that native plants can exhibit stronger constitutive physical defense than invasive species, including greater leaf toughness, while native species may also exhibit more conservative resource-use strategies that favor tissue safety over rapid resource acquisition. These contrasting findings suggest a potential trade-off between investment in carbon-intensive structural defense and the maintenance of more acquisitive growth and metabolic strategies. Such differences indicate that native and invasive species may adopt distinct strategies in allocating carbon to structural components under environmental stress. In contrast, warming effects on structural carbohydrates remain less consistent and are often mediated by interactions with other stressors, particularly water availability.

Chemical defense represents another major carbon sink that interacts with growth and structural investment. Phenolic compounds play central roles in herbivory deterrence and oxidative stress mitigation [21]. Both drought and herbivory can increase phenolic production, but responses are shaped by trade-offs in carbon allocation [8,22]. Comparative studies show that invasive species may differ from native species in defense strategies; for instance, invasive *Ludwigia* species exhibited reduced investment in phenolics and lignin relative to growth [23], whereas invasive *Solidago* species maintained both growth and defense through higher photosynthetic capacity [24]. These contrasting responses indicate that invasive plants do not follow a single growth–defense strategy. Recent evidence further shows that increased tolerance to abiotic stress can occur at the cost of reduced resistance to herbivory in invasive plants, demonstrating that adaptation to one stress may constrain responses to others [25,26]. On the contrary, some invasive plants may mitigate such trade-offs when defensive compounds also contribute to responses to abiotic stress [26]. Under combined stress, defense allocation may be constrained by competing demands for carbon, resulting in non-additive responses [22]. Thus, whether chemical defense is maintained, enhanced, or constrained under multiple stressors likely depends on how plants balance carbon demands associated with growth, herbivore resistance, and abiotic-stress tolerance [27]. These findings highlight that chemical defense must be interpreted within the broader context of carbon allocation trade-offs.

Physiological traits and mobile carbon pools further integrate plant responses to environmental stress. Chlorophyll concentration reflects photosynthetic capacity and typically declines under drought and heat stress due to impairment of photosynthetic systems [28]. However, invasive species often maintain higher photosynthetic performance under stress compared to native species [29], although this advantage is not consistent across physiological traits or environmental conditions. For example, higher photosynthetic rates in invasive species have not always been accompanied by higher water-use efficiency [30], and some invasive species show greater reductions in growth and survival under drought than native species [31,32]. Similarly, invasive and native congeners can exhibit comparable growth and photosynthetic responses to altered resource conditions, indicating that physiological responses are strongly species- and context-dependent [33]. In contrast, soluble sugars accumulate under water limitation, contributing to osmotic adjustment and metabolic stability [34]. Elevated soluble sugar concentrations have been observed in invasive species such as *Ailanthus altissima* and *Triadica sebifera*, supporting stress tolerance and continued growth under drought [10,11]. Nevertheless, higher soluble-carbon concentrations should not be interpreted as a universal feature of invasive plants, because carbon allocation can vary between growth, storage, and stress-tolerance functions depending on species and environmental conditions. Together, these physiological responses reflect shifts between carbon acquisition and carbon conservation and may differ in their magnitude and direction between native and invasive species.

*S. canadensis* (Canada Goldenrod) *and S. decurrens* (Asian Goldenrod) are perennial goldenrods belonging to the family Asteraceae. *S. canadensis* is a widely distributed invasive species in eastern China that frequently forms dense stands in disturbed and semi-natural habitats, where it can displace native species and alter community structure [35]. In contrast, *S. decurrens*, a native congener occupying overlapping habitats within the same climatic region, often co-occurs with the invader but typically exhibits lower dominance and competitive ability. Despite growing evidence indicating that invasive and native plants differ in carbon allocation and stress responses, previous studies have revealed contrasting strategies in growth, physiology, structure, and defense, leaving it unclear how these carbon pools are coordinated under simultaneous abiotic and biotic stressors [27,28]. Here, we compared the invasive *S. canadensis* and its native congener *S. decurrens* in a full-factorial experiment manipulating temperature, soil water availability, and herbivory. We quantified total biomass and root-to-shoot ratio, cell-wall components (hemicellulose, cellulose, and lignin), total phenolic content, chlorophyll concentration, and soluble sugars to evaluate how structural, defensive, and mobile carbon pools respond to combined environmental drivers. The objectives of this study were: (1) to determine which stressor, drought, warming, or herbivory, plays the dominant role in restructuring plant carbon allocation across multiple functional pools; (2) to clarify whether invasive *S. canadensis* and its native *S. decurrens* congeners differ in their carbon allocation strategies, particularly whether *S. canadensis* maintains higher growth and mobile carbon pools while *S. decurrens* shows stronger structural reinforcement; and (3) to examine whether combined drought and herbivory generate trade-offs between growth, defense, and structural investment, and whether these trade-offs differ between invasive *S. canadensis* and native *S. decurrens*. By focusing on three key questions—stressor dominance, invasive–native allocation strategies, and carbon allocation trade-offs under combined stress, this study provides an integrated framework for understanding how interacting global change factors regulate plant carbon economy in invasive and native congeners. It also addresses the unresolved issue of whether contrasting invasive–native stress responses arise from coordinated differences in carbon allocation across multiple competing functional pools.

## 2. Results

### 2.1. Biomass Production and Allocation

**Figure 1:**
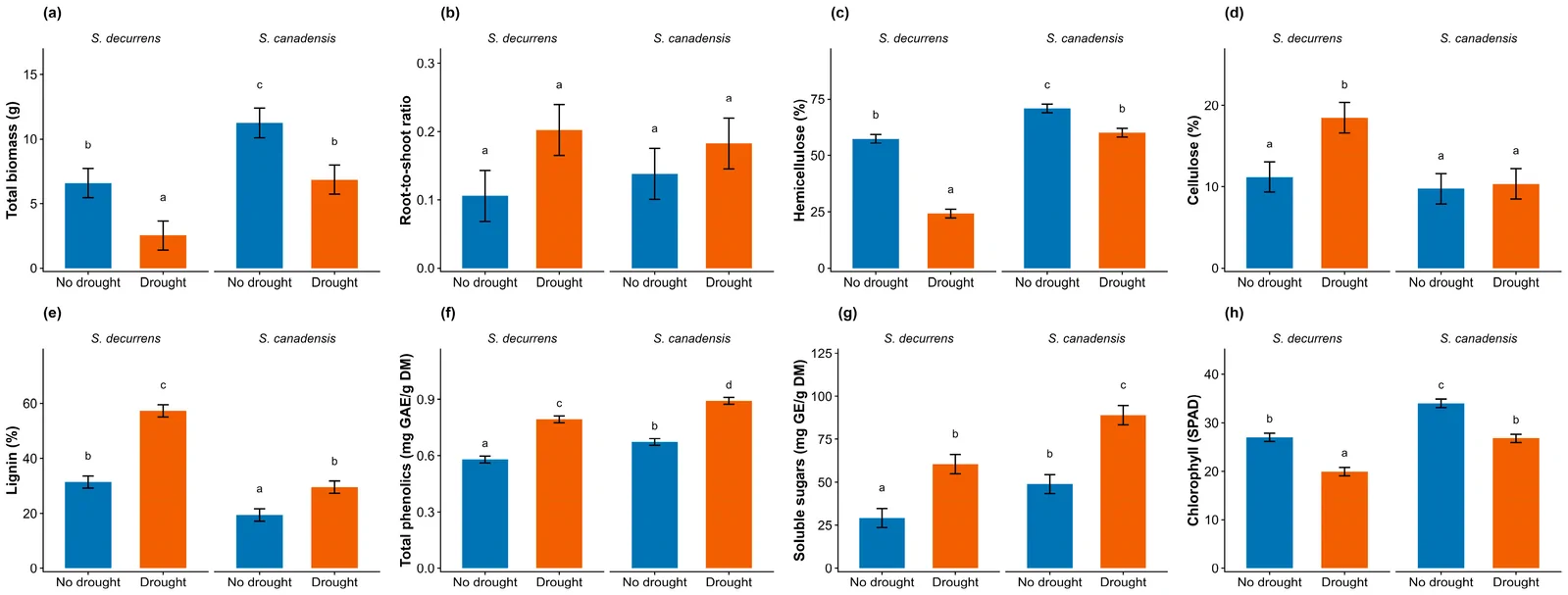
Effects of drought on growth, carbon allocation, structural carbohydrates, defense, and physiological traits in *S. decurrens* and *S. canadensis*. Panels show the effects of drought on (**a**) total biomass, (**b**) root-to-shoot ratio (RSR), (**c**) hemicellulose, (**d**) cellulose, (**e**) lignin, (**f**) total phenolic content (TPC), (**g**) soluble sugars, and (**h**) chlorophyll. Bars represent estimated marginal means ± SE from linear mixed-effects models. Different lowercase letters indicate significant differences among treatment means (Tukey-adjusted, *p* < 0.05).

**Figure 2:**
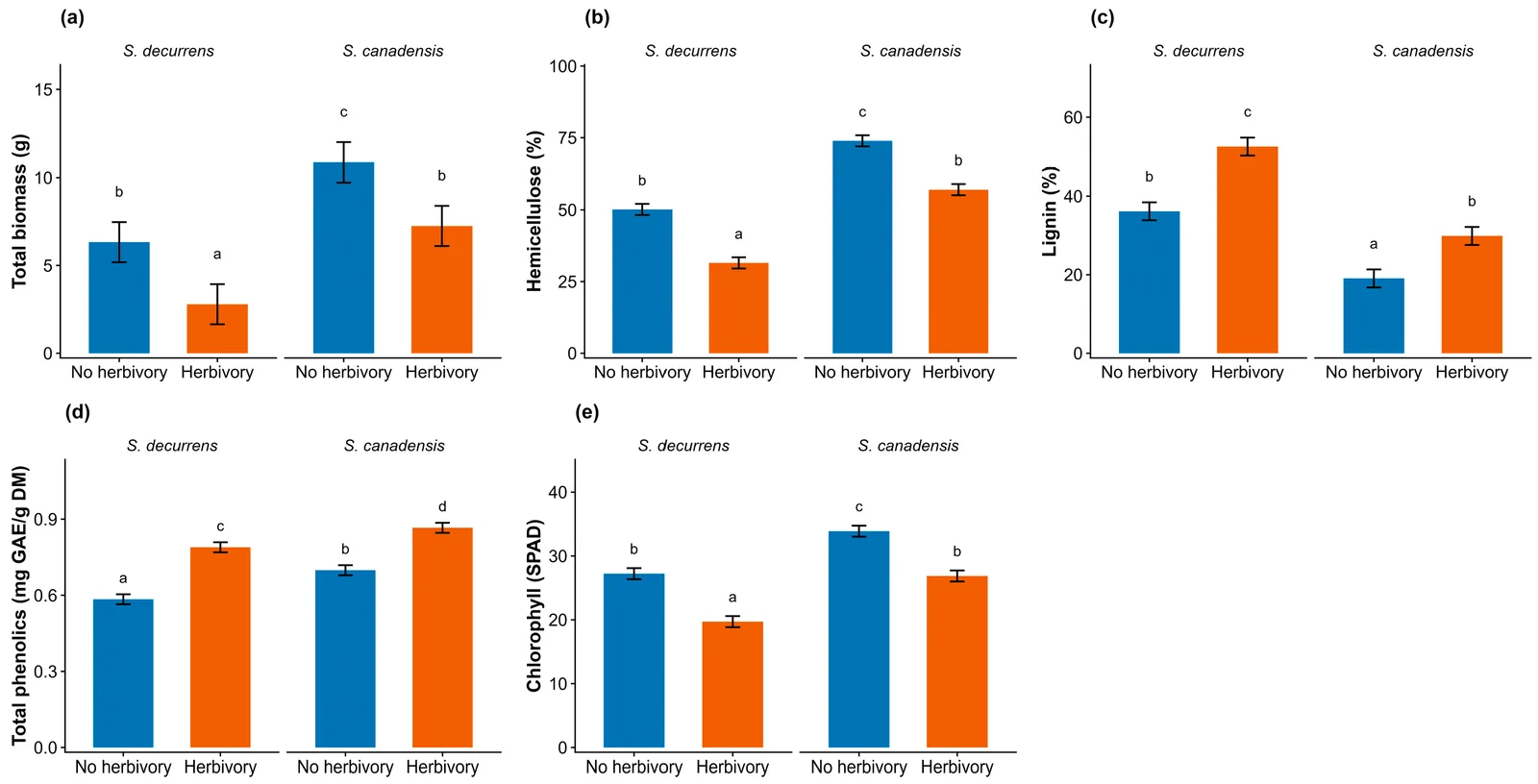
Effects of herbivory on growth, structural carbohydrates, defense, and physiological traits in *S. decurrens* and *S. canadensis*. Panels show the effects of herbivory on (**a**) total biomass, (**b**) hemicellulose, (**c**) lignin, (**d**) total phenolic content (TPC), and (**e**) chlorophyll. Bars represent estimated marginal means ± SE from linear mixed-effects models. Different lowercase letters indicate significant differences among treatment means (Tukey-adjusted, *p* < 0.05).

The total biomass of invasive *S. canadensis* was significantly higher by 98.6% than that of the native *S. decurrens* (Figure S1a, Table 1). Drought significantly reduced total biomass by 47.4% (Figure 1a, Table 1), while herbivory significantly reduced biomass by 41.6% (Figure 2a, Table 1). Warming had no significant effect on total biomass (Table 1). No significant interactions were detected for total biomass (Table 1).

**Figure 3:**
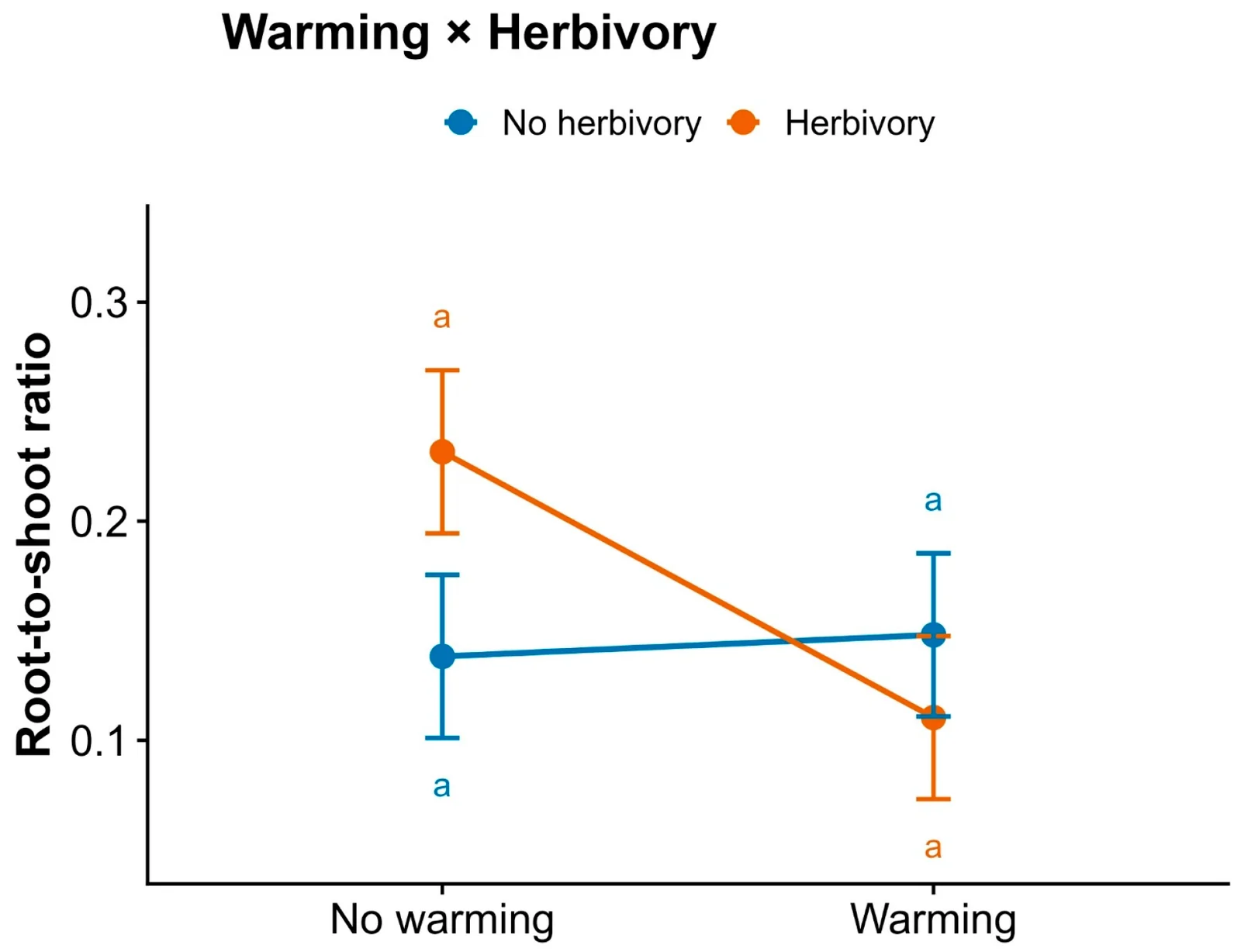
Interactive effects of warming and herbivory on root-to-shoot ratio, with estimated marginal means ± SE for plants under no-herbivory and herbivory treatments at ambient and warmed conditions. Lowercase letters indicate significant differences among treatment means (Tukey-adjusted, *p* < 0.05).

Drought significantly increased root-to-shoot ratio (RSR) by 19.3% (Figure 1b, Table 1). Herbivory increased RSR by 26.6% under control conditions but reduced RSR by 8.9% under the combined warming and herbivory treatment (Figure 3, Table 1).

### 2.2. Structural Carbohydrate Fractions

**Figure 4:**
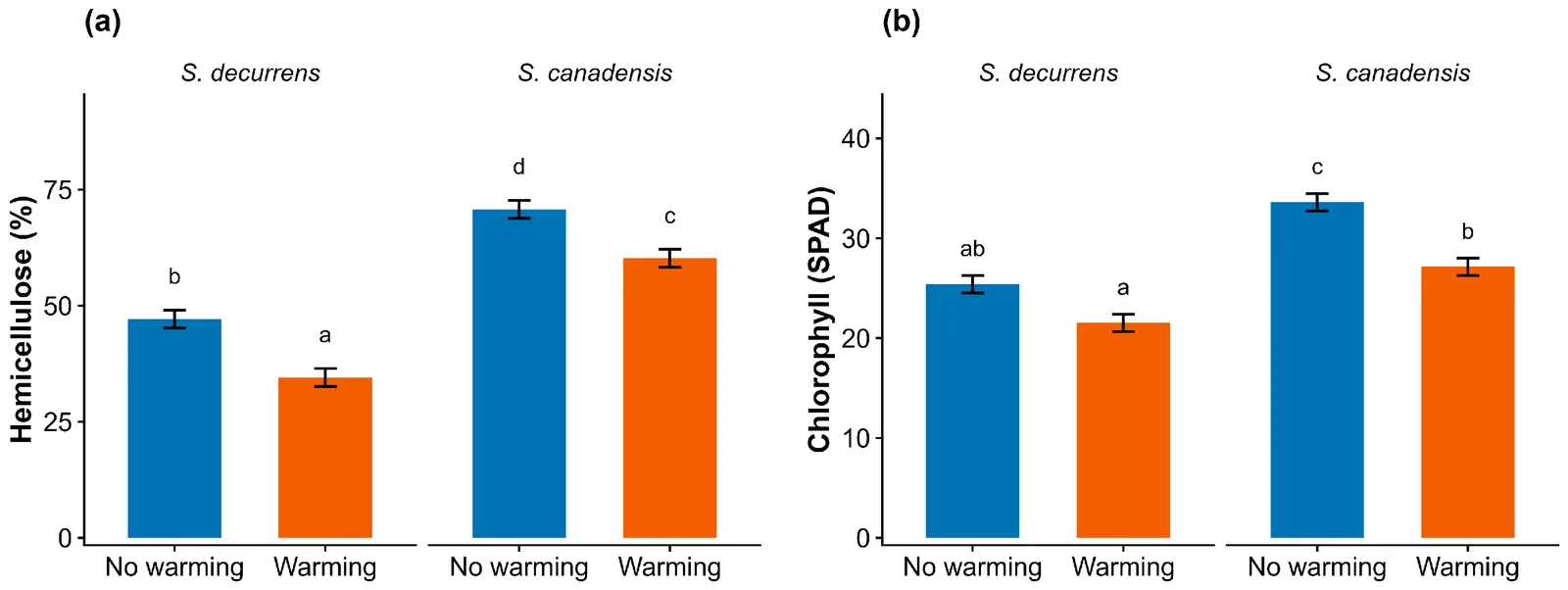
Effects of warming on structural carbohydrate and physiological traits in *S. decurrens* and *S. canadensis*. Panels show the effects of warming on (**a**) hemicellulose and (**b**) chlorophyll. Bars represent estimated marginal means ± SE from linear mixed-effects models. Different lowercase letters indicate significant differences among treatment means (Tukey-adjusted, *p* < 0.05).

**Figure 5:**
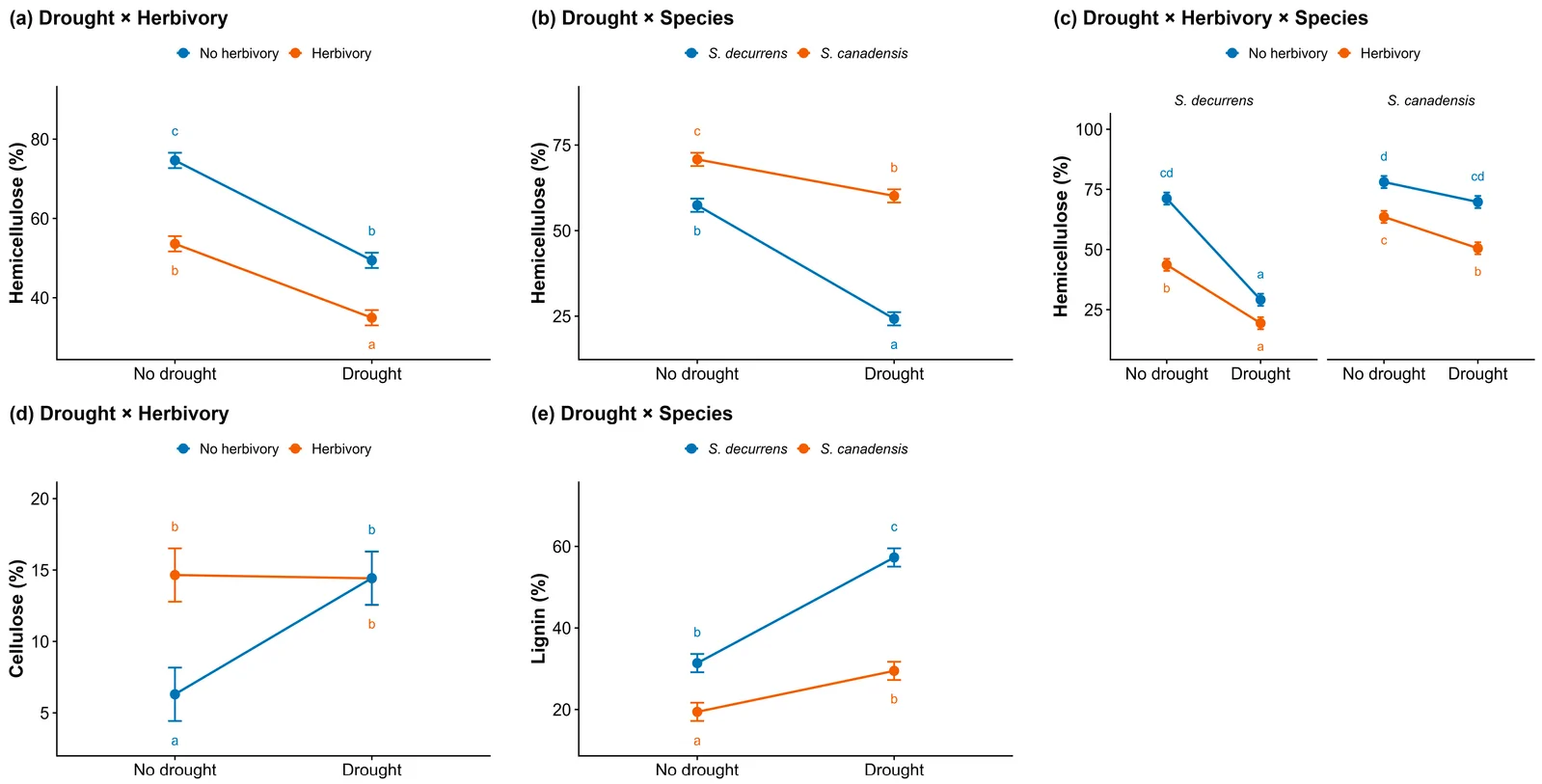
Interactive effects of drought, herbivory, and plant species on structural carbohydrate concentrations. Panels show (**a**) the drought × herbivory interaction for hemicellulose, (**b**) the drought × plant species interaction for hemicellulose, (**c**) the drought × herbivory × plant species interaction for hemicellulose, (**d**) the drought × herbivory interaction for cellulose, and (**e**) the drought × plant species interaction for lignin. Points represent estimated marginal means ± SE from linear mixed-effects models. Different lowercase letters indicate significant differences among treatment means (Tukey-adjusted, *p* < 0.05).

Hemicellulose concentration was 60.4% higher in invasive *S. canadensis* than in native *S. decurrens* (Figure S1b; Table 2). Warming, drought, and herbivory each significantly reduced hemicellulose by 19.6%, 34.2%, and 28.6%, respectively (Figure 4a, Figure 1c, and Figure 2b; Table 2). The drought × herbivory interaction (Table 2) showed that drought reduced hemicellulose more strongly in the absence of herbivory (25.20 vs. 18.65; Figure 5a). The drought × plant species (*S. canadensis* vs. *S. decurrens*) interaction (Table 2) revealed that drought caused a greater reduction in native *S. decurrens* (57.8%) than in invasive *S. canadensis* (15.1%; Figure 5b). A three-way interaction (drought × herbivory × plant species (*S. canadensis* vs. *S. decurrens*), Table 2) further indicated that hemicellulose declined by 55–59% in the native *S. decurrens* and by only 10–21% in the invasive *S. canadensis* under combined treatments (Figure 5c).

Cellulose increased by 37.7% under drought (Figure 1d; Table 2). Native *S. decurrens* had higher cellulose than invasive *S. canadensis* (Figure S1c; Table 2). A drought × herbivory interaction (Table 2) showed that drought increased cellulose by 129.1% in the absence of herbivory but had no effect when herbivory was present (Figure 5d).

Lignin increased under drought (70.7%) and under herbivory (49.2%) (Figure 1e and Figure 2c; Table 2). Native *S. decurrens* had higher lignin than invasive *S. canadensis* (Figure S1d; Table 2). A drought × plant species (*S. canadensis* vs. *S. decurrens*) interaction (Table 2) revealed that the drought-induced increase in lignin was greater in the native *S. decurrens* (82.5%) than in the invasive *S. canadensis* (51.7%; Figure 5e). Warming had no significant effect on either cellulose or lignin (Table 2).

### 2.3. Total Phenolic Content

**Figure 6:**
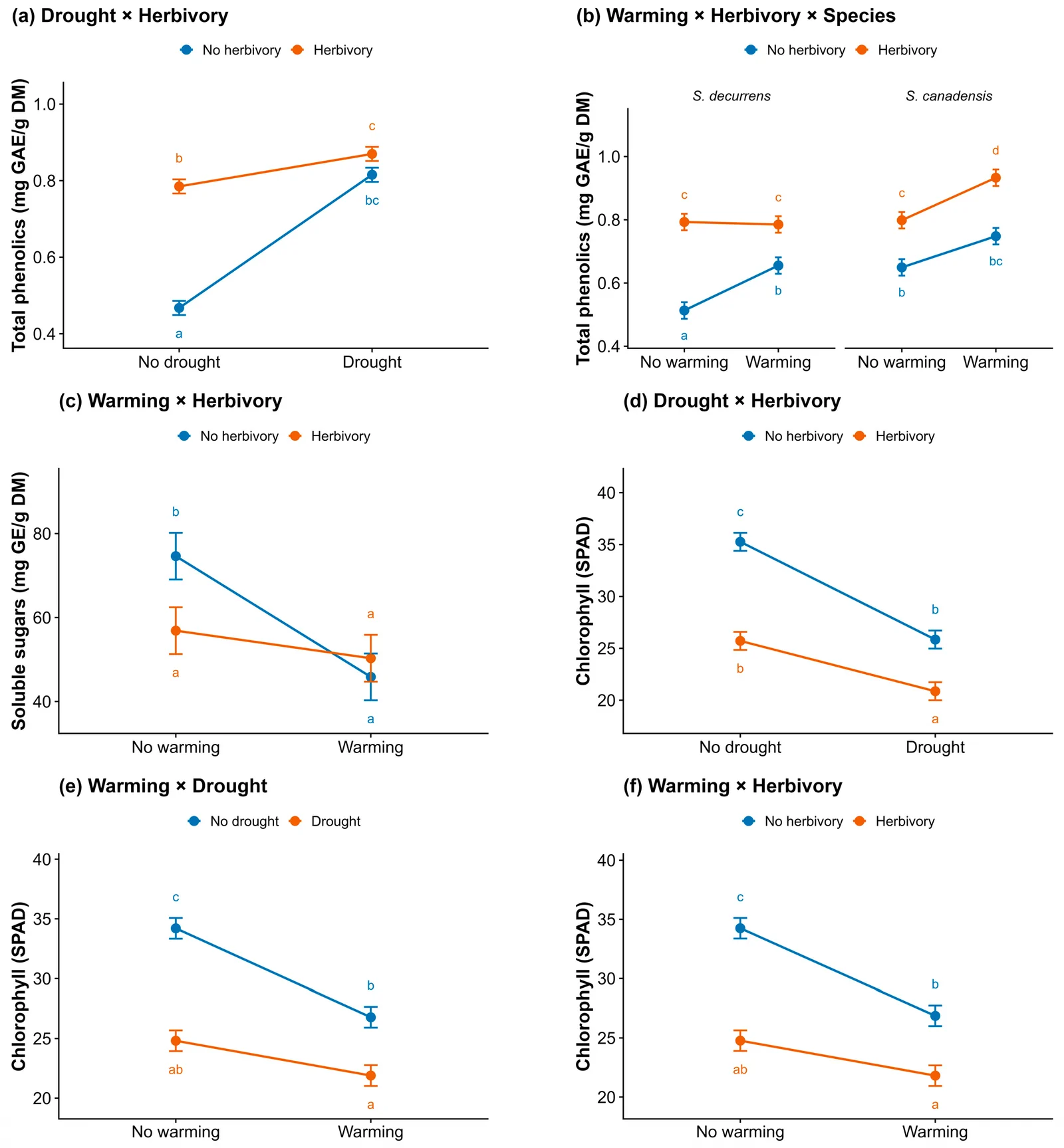
Interactive effects of warming, drought, herbivory, and plant species on defensive and physiological traits. Panels show (**a**) the drought × herbivory interaction for total phenolic content (TPC), (**b**) the warming × herbivory × plant species interaction for TPC, (**c**) the warming × herbivory interaction for soluble sugars, (**d**) the drought × herbivory interaction for chlorophyll, (**e**) the warming × drought interaction for chlorophyll, and (**f**) the warming × herbivory interaction for chlorophyll. Points represent estimated marginal means ± SE from linear mixed-effects models. Different lowercase letters indicate significant differences among treatment means (Tukey-adjusted, *p* < 0.05).

Total phenolic content (TPC) was higher in invasive *S. canadensis* than in native *S. decurrens* (Figure S1e; Table 3). Drought increased TPC by 74.4%, and herbivory also increased TPC (Figure 1f and Figure 2d; Table 3). The effect of drought on TPC depended on herbivory (drought × herbivory interaction, Table 3): drought increased TPC by 74.4% in the absence of herbivory, but only by 10.8% when herbivory was present (Figure 6a). Higher-order interactions involving warming were also significant (Table 3). The warming × herbivory × plant species (*S. canadensis* vs. *S. decurrens*) interaction (Figure 6b) showed that warming increased TPC by 15–28% in invasive *S. canadensis* under herbivory, whereas in native *S. decurrens*, the change was minimal (1.0%). The four-way interaction (warming × drought × herbivory × plant species (*S. canadensis* vs. *S. decurrens*), (Table 3) further revealed that under ambient conditions without herbivory, drought increased TPC by 156.4% in the native *S. decurrens* and by 56.0% in the invasive *S. canadensis*. In contrast, when herbivory was present, these increases were greatly reduced: only 1.2% in the native *S. decurrens* and 19.6% in the invasive *S. canadensis*.

### 2.4. Physiological Indicators of Carbon Status

Soluble sugar concentration was higher in invasive *S. canadensis* than in native *S. decurrens* (Figure S1f; Table 3). Drought increased soluble sugar concentration by 53.6% (Figure 1g; Table 3). The effect of warming on soluble sugars depended on herbivory (warming × herbivory interaction, Table 3). In the absence of herbivory, warming reduced soluble sugars by 32.4%; under herbivory, the reduction was only 6.0% (Figure 6c). Conversely, herbivory reduced soluble sugars by 20.0% under ambient temperature, but increased them by 7.4% under warming (Figure 6c).

Chlorophyll concentration was higher in invasive *S. canadensis* than in native *S. decurrens* (Figure S1g; Table 3). Warming, drought, and herbivory each reduced chlorophyll by 12.2%, 16.2%, and 16.4%, respectively (Figure 4b, Figure 1h, and Figure 2e; Table 3). Interaction of drought × herbivory, warming × drought, and warming × herbivory were significant for chlorophyll (Figure 6d–f, Table 3).

### 2.5. Structural Equation Model Analysis

**Figure 7:**
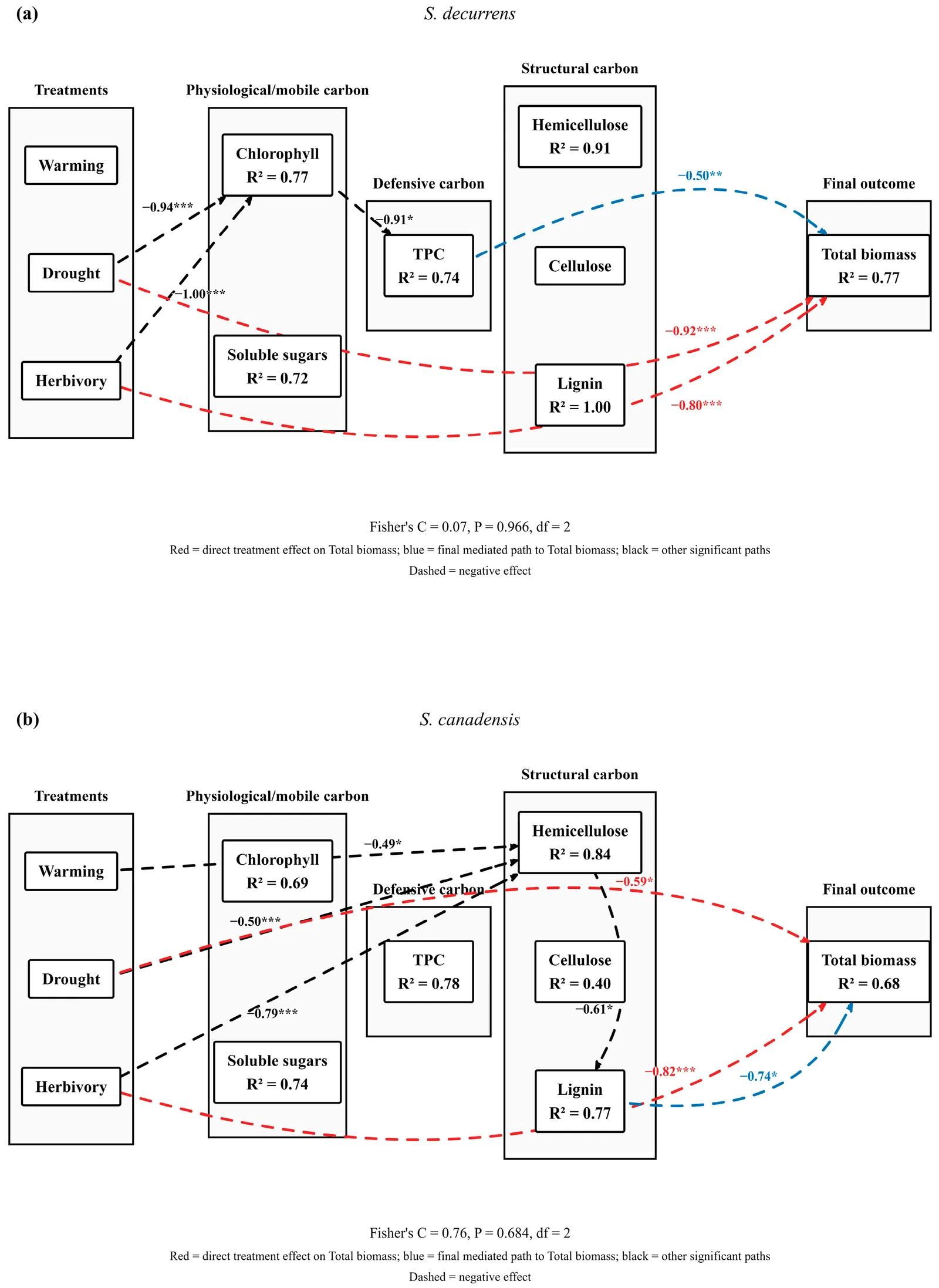
Piecewise structural equation models illustrating the direct and indirect effects of warming, drought, and herbivory on physiological, defensive, and structural carbon traits and their influence on total biomass in (**a**) native (*S. decurrens*) and (**b**) invasive (*S. canadensis*) plants. Red arrows indicate direct treatment effects on total biomass, blue arrows indicate the final trait-mediated pathways to total biomass, and black arrows indicate other significant pathways. Dashed arrows represent negative relationships. Numbers beside arrows are standardized path coefficients, and values inside boxes are marginal R^2^ values. *, **, and *** indicate significance with *p* < 0.05, *p* < 0.01, and *p* < 0.001. Model fit statistics are shown for each model (Fisher’s C, df, and *p* value).

Separate structural equation models showed acceptable fits for both native *S. decurrens* (Fisher’s C = 0.07, df = 2, *p* = 0.966) and invasive *S. canadensis* (Fisher’s C = 0.76, df = 2, *p* = 0.684; Figure 7). For native *S. decurrens*, the model explained 77.1% of the variation in chlorophyll, 71.7% in soluble sugars, 73.7% in total phenolic content (TPC), 90.6% in hemicellulose, 100.0% in lignin, and 76.8% in total biomass. For invasive *S. canadensis*, the model explained 68.6% of the variation in chlorophyll, 74.0% in soluble sugars, 77.9% in TPC, 84.2% in hemicellulose, 39.5% in cellulose, 76.9% in lignin, and 68.5% in total biomass. Across both plant species (*S. canadensis* vs. *S. decurrens*), drought and herbivory significantly affected chlorophyll, hemicellulose, lignin, and total biomass, with additional effects on soluble sugars and TPC, whereas warming significantly reduced hemicellulose only in invasive plants. Trait relationships differed between *S. decurrens* and *S. canadensis*, indicating species-specific relationships among physiological, defensive, and structural carbon traits. In *S. decurrens*, chlorophyll was negatively associated with TPC (β = −0.91), indicating a potential trade-off between photosynthetic function and phenolic defense. No other significant trait-to-trait pathways were detected, although TPC was negatively associated with total biomass (β = −0.50). In *S. canadensis*, chlorophyll was negatively associated with hemicellulose (β = −0.49), TPC was negatively associated with hemicellulose (β = −0.50), and hemicellulose was negatively associated with lignin (β = −0.61), indicating potential trade-offs between physiological, defensive, and structural carbon allocation. Lignin was also negatively associated with total biomass (β = −0.74). No significant positive trait-to-trait pathways were detected in either species (Figure 7; Table S1).

## 3. Discussion

Our results demonstrate that drought is the dominant driver of carbon reallocation, initiating a shift away from aboveground growth toward root investment, structural reinforcement, osmotic adjustment, and defense. Herbivory further modified these drought responses by imposing additional carbon demands that altered the balance between plant defense and growth, and the structural equation models supported these findings by demonstrating direct effects of drought and herbivory on chlorophyll, soluble sugars, total phenolic compounds, structural carbohydrates, and ultimately total biomass. Warming exerts comparatively weaker and context-dependent effects; the SEM indicated that warming influenced relatively few pathways, suggesting that moderate temperature increases alone do not strongly alter carbon allocation unless accompanied by other stressors. These results support the physiological and biochemical responses observed throughout the study, indicating that drought and herbivory, rather than warming, are the principal drivers of coordinated carbon allocation. Importantly, although both species exhibited broadly similar directional responses to stress, the invasive species maintained greater flexibility in carbon allocation by sustaining growth, soluble sugar reserves, and defense capacity under combined stress, as reflected by differences in carbon allocation. In contrast, the native species invested proportionally more carbon in structural stability at the expense of metabolic flexibility, providing a mechanistic explanation for the superior performance of the invasive species under simultaneous abiotic and biotic stress.

### 3.1. Drought Is the Primary Driver of Carbon Reallocation

Among all experimental factors, drought consistently exerted the strongest effects on biomass accumulation, biomass partitioning, structural carbon components, defense compounds, and physiological traits. Drought significantly reduced total biomass while increasing RSR, cellulose, lignin, phenolics, and soluble sugars, accompanied by reductions in chlorophyll concentration. These coordinated responses indicate a major shift in carbon allocation away from aboveground growth and toward survival-related functions, including belowground resource acquisition, structural reinforcement, osmotic regulation, and stress defense. Such drought-induced reallocation patterns are widely recognized as adaptive responses that enhance plant survival under water limitation [36]. Increased RSR under drought likely reflects enhanced investment in root systems to improve water uptake [37], whereas elevated lignin and cellulose contents strengthen cell walls and improve resistance to hydraulic collapse. The accumulation of soluble sugars may contribute to osmotic adjustment and cellular protection during dehydration stress, while increased phenolic production can enhance antioxidant defense and mitigate oxidative damage caused by drought-induced stress [38].

The species-specific responses to drought observed in the present study may partly reflect differences in the ecological associations and drought-response strategies of *S. canadensis* and *S. decurrens*. *S. canadensis* is frequently associated with relatively moist environments and increasingly invades riparian habitats, where it can even occur under waterlogged conditions [39]. Consistent with this moisture association, its biomass production was higher under intermediate water availability than under either drought or waterlogged conditions [39], while drought has been shown to reduce its growth and multiple root and leaf functional traits and may limit its distribution in relatively dry regions [40]. Although *S. canadensis* can acclimate to drier conditions by increasing resistance to drought-induced embolism, this response is accompanied by reduced hydraulic efficiency, indicating a trade-off between hydraulic safety and efficiency [41]. In comparison, experimental work including *S. decurrens* and other native congeners found that native species experienced a smaller proportional reduction in biomass under drought than invasive species and substantially increased their relative allocation to roots under water limitation [42]. This pattern is consistent with the pronounced species-specific allocation response observed in our experiment, in which drought induced a substantially greater increase in RSR in *S. decurrens* than in *S. canadensis*. Together, these findings suggest that the contrasting drought responses of the two species may arise from differences in their strategies for coping with water limitation: *S. canadensis* appears better associated with relatively moist conditions while retaining some capacity for physiological drought acclimation, whereas *S. decurrens* exhibited stronger plastic adjustment of biomass allocation toward belowground resource acquisition under drought. Such differences may therefore contribute to the contrasting carbon-allocation responses of the invasive and native species under water limitation.

In contrast, warming alone had relatively limited independent effects on most measured traits. Significant warming effects appeared primarily through interactions with drought or herbivory, suggesting that moderate warming (+3 °C) may not have exceeded the thermal tolerance range of either species. Nevertheless, warming can indirectly intensify drought stress by increasing evaporative demand and transpiration, thereby accelerating plant water loss and potentially reducing stomatal conductance and photosynthetic carbon assimilation under water limitation. In the present study, such temperature–water coupling was trait-dependent: a significant warming × drought interaction occurred for chlorophyll, whereas this interaction was not significant for biomass, RSR, or the measured carbon-allocation traits. Instead, water limitation likely overrode warming effects, consistent with previous studies showing that drought often exerts stronger constraints on plant performance than moderate temperature increases [43]. The lack of broader warming × drought interactions may therefore reflect the dominant effect of the imposed drought treatment (30–50% WHC), which could have limited the additional detectable influence of moderate warming on carbon allocation. This finding emphasizes that under future climate scenarios, precipitation variability may play a more decisive role than moderate warming in regulating plant carbon allocation.

Herbivory independently reduced biomass and chlorophyll content while increasing phenolics and lignin, indicating activation of defensive and repair-related processes. However, its most important role emerged through interactions with drought. Although drought alone strongly increased total phenolics, the addition of herbivory reduced this response. Similar antagonistic patterns were observed in the higher-order interactions involving warming, drought, herbivory, and invasion status [44]. These results suggest that plants under combined stressors face competing carbon demands. Under drought alone, plants can allocate more carbon to the production of defensive compounds such as phenolics. When herbivory occurs simultaneously, carbon must also be allocated to tissue repair, regrowth, and maintenance of damaged tissues, reducing the resources available for additional defense production [25,45]. This interpretation is consistent with the theory that carbon allocation under multiple stress conditions is governed not only by resource availability but also by prioritization among competing physiological functions.

### 3.2. S. canadensis vs. S. decurrens Exhibit Contrasting Allocation Strategies

Although both invasive *S. canadensis* and native *S. decurrens* displayed similar directional responses to drought, the magnitude and balance of their allocation shifts differed substantially. Across treatments, the invasive species maintained higher biomass, higher chlorophyll concentration, greater soluble sugar accumulation, and higher hemicellulose content under drought compared with the native species. In contrast, the native species exhibited stronger increases in lignin and cellulose under stress. These contrasting responses suggest that the two species occupy different positions along a continuum of carbon allocation strategies. The invasive *S. canadensis* appears to adopt a flexible, growth-oriented strategy that simultaneously maintains multiple functional carbon sinks. However, because the present study examined only one invasive–native congeneric pair, this greater allocation flexibility should be interpreted as a species-specific difference between *S. canadensis* and *S. decurrens*, rather than as a general pattern of invasive versus native plants. Comparative evidence indicates that invasive–native differences are context-dependent; although meta-analyses have reported stronger performance of invasive species than native species under some environmental changes, including drought, the magnitude and direction of these differences vary among species and environmental conditions. Bradbury and Hofstra Bradbury and Hofstra [46] demonstrated that a high proportion of assimilated carbon is allocated to leaves and stems during early growth, while Zhang, Xu Zhang, Xu [47] documented a trade-off whereby introduced populations allocate more resources to growth (via highly lignified xylem) but less to defense compared to native populations. Additionally, *S. canadensis* exhibits phenotypic plasticity under salinity and drought stress [48]. Higher soluble sugar concentrations likely provide a readily available carbon reserve that supports continued metabolic activity during stress [10]. In addition, the relatively smaller reduction in hemicellulose under drought may preserve cell wall flexibility and facilitate recovery after stress release [49].

By comparison, the native *S. decurrens* appears to favor a more conservative strategy characterized by stronger structural reinforcement through lignification and cellulose accumulation. While this strategy may improve tissue stability and drought resistance, it is likely to occur at the expense of growth and physiological flexibility. Such patterns align with the plant economics spectrum framework, which proposes a trade-off between resource acquisition and resource conservation strategies. However, our findings further extend this framework by demonstrating that the contrasting responses of *S. canadensis* and *S. decurrens* may depend on how carbon is partitioned among structural, metabolic, and defensive functions under multiple simultaneous stressors [50]. Because only one congeneric species pair was examined, species-specific functional differences cannot be fully separated from differences associated with invasion status. Therefore, broader conclusions regarding invasive and native plants require validation across multiple independent species pairs. Although *S. canadensis* seeds were sampled from multiple natural populations, they were used without a refresher generation. Therefore, potential maternal environmental effects cannot be excluded and may have accounted for some of the observed differences between the two species.

Several limitations should be considered when interpreting the findings of this study. First, the experiment was conducted under controlled greenhouse conditions over a single growing period; therefore, the observed responses may differ under longer-term field conditions where environmental factors are more variable. Second, herbivory was represented by a single herbivore species, Spodoptera litura, and the responses observed here are specific to the herbivory treatment examined. Third, the warming treatment represented a fixed increase of +3 °C, and conclusions regarding warming are therefore restricted to the experimental conditions applied in this study. Fourth, the comparison was limited to one congeneric species pair, *S. canadensis* and *S. decurrens*. Consequently, the observed differences should be interpreted specifically for these two species rather than generalized to invasive and native plants as a whole. In addition, because *S. canadensis* was established directly from field-collected seeds without an intermediate refresher generation, potential maternal environmental effects cannot be completely excluded. Future studies involving multiple invasive–native species pairs, longer experimental periods, different herbivory regimes, and a broader range of warming conditions will be important for evaluating the generality of the patterns identified here.

## 4. Materials and Methods

### 4.1. Study Site

The study was conducted at the controlled warm-climate garden of the Jiaojiang Campus, Taizhou University, Zhejiang Province, China (28.6556° N, 121.3851° E; approximately 10 m above sea level). The region experiences a subtropical monsoon climate with mean annual temperatures of 15–20 °C and annual precipitation ranging from 1000 to 2000 mm (https://www.zjtz.gov.cn/col/col1229049312/index.html; accessed on 20 August 2024).

### 4.2. Seeds Germination and Transplanting

A congeneric species pair comprising the invasive *Solidago canadensis* and the native *S. decurrens* was used in this study. In November 2023, seeds of *S. canadensis* were collected from multiple natural populations near the Jiaojiang Campus, pooled, air-dried, and stored in paper envelopes under cool, dry, and dark conditions until use. The field-collected seeds were used directly to establish the experimental plants without an intermediate refresher generation under common environmental conditions. In mid-June 2024, seeds of *S. canadensis* were sown in trays containing peat moss and germinated under controlled conditions in the greenhouse.

On 7 July 2024, seedlings of *S. decurrens* originating from the same geographic region as the *S. canadensis* populations were obtained from Tujia Yaoxiang Seedling Company (Lishui City, China). Seedlings were immediately processed upon receipt, gently separated, and roots were inspected and cleaned to remove excess substrate. Damaged or necrotic tissues were trimmed where necessary. The seedlings were then transplanted into trays containing peat-based substrate and maintained under the same greenhouse conditions as *S. canadensis* seedlings to allow recovery and acclimation before experimental transplanting; on 29 July 2024, only healthy seedlings of similar height from both species were selected to reduce initial source-related variation. Seedling selection was conducted immediately before transplantation using the same morphological criteria for both species, with individuals showing abnormal growth, visible damage, or substantial differences in size excluded. This standardized selection, together with the common acclimation conditions, was used to minimize initial variation between the two species before the experimental treatments were performed.

The topsoil was collected from a mountainous area near Taizhou City and was slightly acidic (pH 4.53), with organic matter content of 11.71 g kg^−1^, total nitrogen of 0.280 g kg^−1^, and total phosphorus of 1.206 g kg^−1^. The soil mixture was thoroughly homogenized before being distributed among pots, and all experimental plants were grown in the same soil mixture without differential nutrient supplementation, thereby maintaining consistent initial soil conditions across species and treatment combinations. On 29 July 2024, seedlings of both species with similar height were selected and transplanted into 12 cm diameter × 10.8 cm depth plastic pots filled with a 2:1 sand–topsoil mixture. A total of 48 pots were used in the experiment, including six control pots (three *S. canadensis* and three *S. decurrens*) maintained in a greenhouse under 25 °C/18 °C day–night temperatures, natural light conditions, and approximately 60% relative humidity. Pots were repositioned regularly to minimize spatial bias.

### 4.3. Experimental Design

A full-factorial design was implemented to examine the effects of temperature, water availability, herbivory, and plant species (*S. canadensis* vs. *S. decurrens*). The four fixed factors were: plant species (invasive *S. canadensis* vs. native *S. decurrens*), warming (ambient vs. +3 °C above ambient), water availability (80–90% vs. 30–50% water-holding capacity, WHC), herbivory (absence vs. presence). Each factor had two levels, generating 16 treatment combinations. Three replicate blocks were established, yielding a total of 48 experimental units (2 × 2 × 2 × 2 × 3). The experiment followed a split-plot structure, with warming assigned at the plot level within each block, consistent with previous infrared-heating warming experiments [51]. Within each block, warming was applied at the plot level. Each block contained two temperature plots: one ambient control and one warmed plot.

#### 4.3.1. Simulated Warming Treatment

Warming was achieved using three infrared heating lamps (model LPR2420/1500-L, Longpro Co., Ltd., Guangzhou, China) suspended 2.5 m aboveground. Control plots received no supplemental heating. Air temperature was monitored continuously using DTE10T sensors (Delta Co., Ltd., Taiwan, China) positioned 0.5 m above the canopy, confirming an average temperature increase of approximately +3 °C in the warmed plots relative to the ambient treatments. Within each temperature plot, pots were randomly assigned to drought and herbivory treatments. In total, six main plots (three blocks × two temperature levels) were established, each containing eight pots. The warming treatment was initiated on 20 August 2024, 22 days after transplanting, and was maintained continuously until plant harvest on 8 November 2024.

#### 4.3.2. Drought Treatment

Water treatments commenced on 20 August 2024, 22 days after transplanting, and were maintained until plant harvest on 8 November 2024. Soil moisture was monitored daily using a ProCheck Soil Moisture Meter (METER Group, Inc., Pullman, WA, USA). Pots assigned to drought conditions were maintained at 30–50% WHC, while control pots were maintained at 80–90% WHC. Irrigation frequency was adjusted accordingly, with drought treatments watered every two to three days and controls every one to two days. Pots were placed on trays to minimize evaporative losses, moisture levels were checked throughout the experiment, and irrigation was adjusted when necessary to keep pots within the assigned WHC ranges.

#### 4.3.3. Herbivory Treatment

Herbivory was introduced on 10 October 2024 using two third-instar *Spodoptera litura* larvae per plant [52,53]. Larvae were placed on young apical leaves to promote active feeding. All plants, including non-herbivory controls, were enclosed individually within mesh cages (60 × 60 × 90 cm) to prevent larval escape and maintain consistent microclimatic conditions. Missing or dead larvae were replaced on 17 and 22 October to maintain comparable feeding pressure. Herbivory treatments lasted four weeks and concluded on 8 November 2024. Herbivory was included as a fixed factor in the statistical analyses. However, visual damage scores were not analyzed as a response variable; they were used only to confirm that feeding occurred and to monitor consistency of herbivory application across plants. Feeding damage was visually inspected using a standardized 0–5 damage scale adapted from commonly used *Spodoptera* leaf damage rating systems [54], and all assessments were conducted by the same observer to minimize scoring bias.

#### 4.3.4. Trait Measurements

On 8 November 2024 (after 12 weeks of growth), chlorophyll concentration was measured using a SPAD-502 chlorophyll meter (Konica Minolta sensing, Inc, Sakai, Osaka Japan). Six readings were taken from fully expanded leaves per plant at comparable positions in the upper portion of the canopy. Only mature, healthy, fully expanded leaves were selected, while young expanding, senescent, visibly damaged, and herbivore-damaged leaves were avoided. Measurements were taken from the leaf lamina while avoiding the midrib and major veins. The mean of the six readings was used as the chlorophyll value for each individual. Then, plants were harvested. Plants were separated into shoots and roots. The biomass was measured after being oven-dried at 70 °C for 72 h. Root-to-shoot ratio (RSR) was calculated as root biomass divided by shoot biomass, and biomass fractions were expressed as proportions of total dry mass [53]. Total phenolic content (TPC) was quantified using the Folin–Ciocalteu method. Absorbance was measured at 765 nm using a spectrophotometer, with gallic acid used as the calibration standard. A series of gallic acid solutions of known concentrations was used to construct the standard calibration curve, and sample concentrations were calculated from the resulting calibration relationship. Sample extracts were diluted, when necessary, to ensure that absorbance values fell within the linear range of the gallic acid standard curve. Results were expressed as mg gallic acid equivalents (GAE) per gram dry weight. [21]. Soluble sugars were determined using the anthrone colorimetric method [55], with absorbance measured at 620 nm using a spectrophotometer, with glucose as the standard. A series of glucose solutions of known concentrations was used to construct the standard calibration curve, and soluble sugar concentrations were calculated from the resulting calibration relationship. Sample extracts were diluted, when necessary, to ensure that absorbance values fell within the linear range of the glucose standard curve and concentrations were expressed as mg glucose equivalents per g dry weight (mg GE g^−1^ DW). Structural carbohydrate fractions were determined using the sequential detergent fiber method of Van Soest and Wine [18]. Dried and ground leaf samples were first subjected to neutral detergent extraction to obtain neutral detergent fiber (NDF), followed by acid detergent extraction to obtain acid detergent fiber (ADF), and subsequent sulfuric acid digestion to determine acid detergent lignin (ADL). All detergent solutions, digestion conditions, and analytical procedures were prepared and applied according to the original protocol of Van Soest and Wine [18]. Hemicellulose was calculated as the difference between NDF and ADF, cellulose as the difference between ADF and ADL, and lignin was represented by the ADL fraction. Structural carbohydrate concentrations were expressed as percentages of dry leaf mass.

### 4.4. Statistical Analysis

To test the effects of warming, drought, herbivory, plant species (*S. canadensis* vs. *S. decurrens*), and their interactions on plant traits, linear mixed-effects models were fitted for each response variable. Fixed effects included plant species, warming, drought, herbivory, and all possible interaction terms. To account for the split-plot experimental design, random intercepts were included for Plot nested within Block. Because herbivory treatments were applied within net enclosures, net (Block × Plot × Herbivory) was included as an additional random factor to account for potential non-independence among plants sharing the same enclosure.

To evaluate the contribution of higher-order interactions, a full model containing the four-way interaction (Warming × Drought × Herbivory × plant species) was compared with a reduced model excluding only this interaction term. Likelihood ratio tests were used to assess whether inclusion of the highest-order interaction improved model fit. Final inference was based on the selected model using Type III tests with sum-to-zero contrasts.

Model assumptions were assessed using residual diagnostics, including quantile–quantile plots and residuals versus fitted values. Where necessary, response variables were transformed to improve normality and homoscedasticity.

To evaluate the direct and indirect relationships among experimental treatments, plant species, growth allocation traits, physiological/mobile carbon traits, defensive carbon traits, structural carbon traits, and total biomass, an integrated piecewise structural equation model (SEM) was constructed using the piecewiseSEM package [56]. Individual component models were fitted as linear mixed-effects models, and overall model fit was assessed using Fisher’s C statistic based on tests of directed separation. The explanatory power of each endogenous variable was quantified using marginal R^2^ values.

All statistical analyses were conducted in R [57]. Linear mixed-effects models were fitted using the lme4 package [58], with Kenward–Roger approximation for denominator degrees of freedom implemented via lmerTest [59]. Estimated marginal means and Tukey-adjusted pairwise comparisons were obtained using emmeans [60], and compact letter displays were generated using multcomp [61]. Results are reported as estimated marginal means ± standard error (SE), with α = 0.05.

## 5. Conclusions

This study demonstrates that drought is the primary driver regulating carbon allocation in both invasive and native *Solidago* species, whereas warming plays a comparatively minor and interaction-dependent role. Herbivory further modified drought responses by creating competing carbon demands that reduced investment in defense under combined stress. Although both species exhibited similar directional stress responses, the invasive *S. canadensis* maintained greater flexibility in allocating carbon among growth, defense, and storage functions, while the native *S. decurrens* adopted a more structurally conservative strategy. These findings suggest that invasion success under future climate change may depend less on individual traits and more on the ability to dynamically redistribute carbon among competing physiological processes under multiple simultaneous stressors.

## Figures and Tables

**Table 1:** Linear mixed-effects model results testing the effects of warming, drought, herbivory, plant species (*S. canadensis* vs. *S. decurrens*), and their interactions on total biomass and root-to -shoot ratio (RSR). Fixed effects are presented with numerator and denominator degrees of freedom (NumDF, DenDF), *F* values, and associated *p* values. Block, plot (nested within block), and net identity were included as random effects. NetID represents the identity of the net enclosure (Block × Plot × Herbivore) and accounts for potential non-independence among plants sharing the same enclosure. Standard deviations (SD) of random terms and residual variance are shown. Statistically significant effects are indicated in bold.

Term			Total Biomass		RSR	
Fixed Effect	NumDF	DenDF	*F*	*p*	*F*	*p*
Warming	1	2	0.445	0.573	3.326	0.210
Drought	1	25	67.215	**<0.001**	5.315	**0.029**
Herbivory	1	4	35.969	**0.004**	0.829	0.370
Species	1	25	75.814	**<0.001**	0.042	0.839
Drought:Herbivory	1	25	1.983	0.171	0.116	0.736
Drought:Species	1	25	0.109	0.745	0.716	0.404
Herbivory:Species	1	25	0.005	0.945	0.170	0.683
Warming:Drought	1	25	1.463	0.238	1.852	0.184
Warming:Herbivory	1	4	2.474	0.191	4.604	**0.040**
Warming:Species	1	25	0.865	0.361	1.873	0.182
Drought:Herbivory:Species	1	25	2.459	0.129	0.246	0.624
Warming:Drought:Herbivory	1	25	3.283	0.082	0.521	0.476
Warming:Drought:Species	1	25	0.235	0.632	0.012	0.915
Warming:Herbivory:Species	1	25	0.923	0.346	0.215	0.646
Random effects (SD)			SD		SD	
Block			1.389		0.037	
Block/PlotID			1.456		0.000	
NetID			0.515		0.000	
Residual			1.788		0.106	

**Table 2:** Linear mixed-effects model results for structural traits (hemicellulose, cellulose, and lignin) in response to warming, drought, herbivory, plant species (*S. canadensis* vs. *S. decurrens*), and their interactions. Fixed effects are reported with NumDF, DenDF, *F* values, and *p* values. Random effects included block, plot nested within block, and net identity; NetID represents the identity of the net enclosure (Block × Plot × Herbivore) and accounts for potential non-independence among plants sharing the same enclosure. Standard deviations (SD) and residual variance are provided. Statistically significant effects are indicated in bold.

Term			Hemicellulose		Cellulose		Lignin	
Fixed Effect	NumDF	DenDF	*F*	*p*	*F*	*p*	*F*	*p*
Warming	1	2	43.459	**0.022**	1.128	0.4	16.063	0.057
Drought	1	25	193.604	**<0.001**	4.474	**0.045**	67.506	**<0.001**
Herbivory	1	4	102.586	**0.001**	4.978	0.09	32.367	**0.005**
Species	1	25	244.886	**<0.001**	6.56	**0.017**	82.531	**<0.001**
Drought:Herbivory	1	25	4.320	**0.048**	5.018	**0.034**	0.172	0.682
Drought:Species	1	25	51.049	**<0.001**	3.186	0.086	13.122	**0.001**
Herbivory:Species	1	25	0.320	0.577	1.017	0.323	1.609	0.216
Warming:Drought	1	25	0.252	0.620	3.716	0.065	1.648	0.211
Warming:Herbivory	1	4	0.615	0.477	0.191	0.684	0.055	0.827
Warming:Species	1	25	0.423	0.521	0.016	0.902	0.331	0.57
Drought:Herbivory:Species	1	25	12.819	**0.001**	3.996	0.057	0.761	0.391
Warming:Drought:Herbivory	1	25	0.572	0.457	2.039	0.166	3.11	0.09
Warming:Drought:Species	1	25	0.104	0.750	0.523	0.476	0.722	0.404
Warming:Herbivory:Species	1	25	0.111	0.742	0.306	0.585	0.507	0.483
Random effects (SD)			SD		SD		SD	
Block			1.739		0		0	
Block/PlotID			0		0		0	
NetID			1.332		0		1.662	
Residual			5.458		6.469		7.579	

**Table 3:** Linear mixed-effects model results for defensive and physiological traits (total phenolic content, simple sugars, and chlorophyll) under warming, drought, herbivory, plant species (*S. canadensis* vs. *S. decurrens*), and their interactions. Fixed effects are presented with NumDF, DenDF, *F* values, and *p* values. Block, plot nested within block, and net identity were fitted as random effects. NetID represents the identity of the net enclosure (Block × Plot × Herbivore) and accounts for potential non-independence among plants sharing the same enclosure, and corresponding standard deviations (SD) are reported. Statistically significant effects are indicated in bold.

Term			TPC		Simple Sugars		Chl	
Fixed Effect	NumDF	DenDF	*F*	*p*	*F*	*p*	*F*	*p*
Warming	1	2	15.602	0.059	16.767	0.055	35.327	**0.027**
Drought	1	25	195.147	**<0.001**	68.629	**<0.001**	67.31	**<0.001**
Herbivory	1	4	63.912	**0.001**	2.38	0.134	69.728	**<0.001**
Species	1	25	38.116	**<0.001**	31.202	**<0.001**	63.057	**<0.001**
Drought:Herbivory	1	25	72.126	**<0.001**	0.417	0.523	6.901	**0.014**
Drought:Species	1	25	0.023	0.882	1.033	0.318	0.007	0.932
Herbivory:Species	1	25	1.481	0.235	0.17	0.683	0.081	0.778
Warming:Drought	1	25	0.116	0.736	0.764	0.389	6.714	**0.015**
Warming:Herbivory	1	4	1.505	0.287	6.615	**0.016**	6.456	**0.017**
Warming:Species	1	25	2.544	0.124	1.453	0.238	2.233	0.146
Drought:Herbivory:Species	1	25	3.431	0.076	0.015	0.903	1.349	0.255
Warming:Drought:Herbivory	1	25	0.821	0.374	0.985	0.329	0.459	0.503
Warming:Drought:Species	1	25	0.464	0.502	0.273	0.605	0.002	0.962
Warming:Herbivory:Species	1	25	8.983	**0.006**	2.271	0.143	0.756	0.392
Warming:Drought:Herbivory:Species	1	25	8.871	**0.007**				
Random effects (SD)			SD		SD		SD	
Block			0		6.108		0	
Block/PlotID			0		0		0	
NetID			0.03		0		0	
Residual			0.054		14.942		3.014	

## Data Availability

Data will be made available upon request.

## Supplementary Materials

The following supporting information can be downloaded at: https://www.mdpi.com/article/10.3390/plants15182746/s1, Table S1. Standardized path coefficients (β) and associated *p* values for all significant pathways retained in the separate piecewise structural equation models for native (*S. decurrens*) and invasive (*S. canadensis*) plants. The table includes significant treatment-to-trait and trait-to-trait relationships retained in the final models, including pathways that do not terminate at total biomass. Dashes (–) indicate pathways that were not retained or were not statistically significant in the corresponding model. Asterisks denote significance levels (*p* < 0.05, *p* < 0.01, *p* < 0.001). Figure S1. Differemces in growth, structural carbohydrates, defense and physiological traits between native (*S. decurrens*) and invasive (*S. canadensis*) Panels show species differences in (a) total biomass, (b) hemicellulose, (c) cellulose, (d) lignin, (e) total phenolic content (TPC), (f) soluble sugars and (g) chlorophyll. Bars represent estimated marginal means ± SE. Different lowercase letters indicate significant differences among treatment means (Tukey-adjusted, *p* < 0.05).
