# Supplementary material for: Drought Dominates over Warming in Structuring Growth and Defense in Invasive Solidago canadensis vs. Native Solidago decurrens
Source: Plants (Basel). 2026 Sep 8;15(18):2746. doi: 10.3390/plants15182746 (PMC13610744; doi:10.3390/plants15182746)
Supplement: Supplementary file 1 [file plants-15-02746-s001.zip › plants-4507190-supplementary.pdf]

Supplementary Material

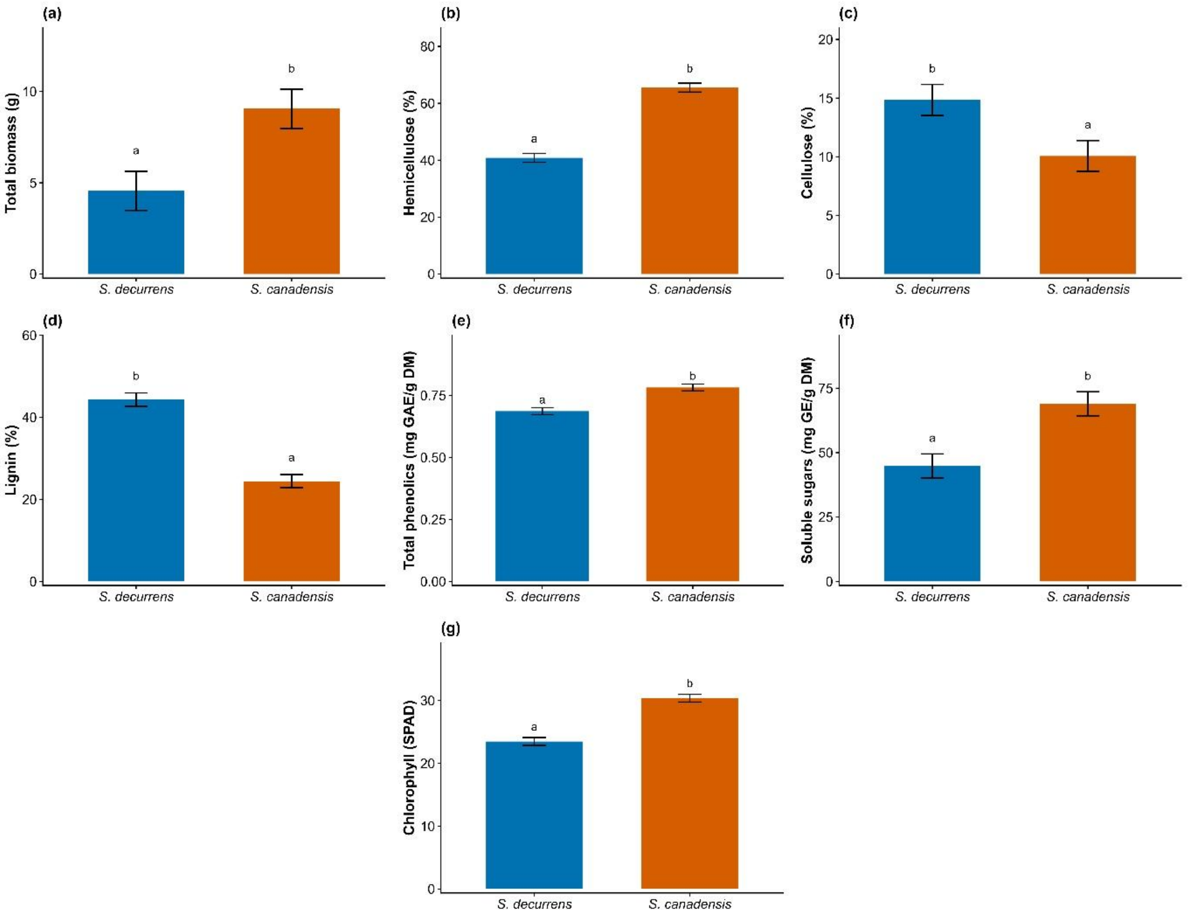

Figure S1. Differemces in growth, structural carbohydrates, defense and physiological traits between native (*S. decurrens*) and invasive (*S. canadensis*) Panels show species differences in (a) total biomass, (b) hemicellulose, (c) cellulose, (d) lignin, (e) total phenolic content (TPC), (f) soluble sugars and (g) chlorophyll. Bars represent estimated marginal means ± SE. Different lowercase letters indicate significant differences among treatment means (Tukey-adjusted, $p < 0.05$).

Table S1. Standardized path coefficients (β) and associated *p* values for all significant pathways retained in the separate piecewise structural equation models for native (*S. decurrens*) and invasive (*S. canadensis*) plants. The table includes significant treatment-to-trait and trait-to-trait relationships retained in the final models, including pathways that do not terminate at total biomass. Dashes (–) indicate pathways that were not retained or were not statistically significant in the corresponding model. Asterisks denote significance levels ($p < 0.05$, $p < 0.01$, $p < 0.001$).

Pathway	Native_coefficient	Native_P	Invasive_coefficient	Invasive_P
Drought -> Chlorophyll	-0.938***	<0.001	-0.958***	<0.001
Herbivory -> Chlorophyll	-0.998***	<0.001	-0.932***	<0.001
Drought -> Soluble sugars	1.099***	<0.001	1.406***	<0.001
Herbivory -> Soluble sugars	-0.296*	0.021	-	-
Drought -> TPC	1.159***	<0.001	1.184***	<0.001
Herbivory -> TPC	1.109***	<0.001	0.905***	<0.001
Warming -> Hemicellulose	-	-	-0.490*	0.041
Drought -> Hemicellulose	-1.544***	<0.001	-0.496***	<0.001
Herbivory -> Hemicellulose	-0.868***	<0.001	-0.785***	<0.001
Herbivory -> Cellulose	-	-	0.765**	0.004
Drought -> Lignin	1.441***	<0.001	0.559***	<0.001
Herbivory -> Lignin	0.911***	<0.001	0.602***	<0.001
Drought -> Total biomass	-0.923***	<0.001	-0.588*	0.01
Herbivory -> Total biomass	-0.804***	<0.001	-0.820***	<0.001
Chlorophyll -> TPC	-0.907*	0.011	-	-
TPC -> Hemicellulose	-0.313*	0.012	-	-
Hemicellulose -> Cellulose	-	-	-1.345*	0.023
Hemicellulose -> Lignin	-1.195***	<0.001	-0.612*	0.014
Cellulose -> Lignin	-0.440***	<0.001	-	-
TPC -> Total biomass	-0.504**	0.001	-	-
Lignin -> Total biomass	-	-	-0.737*	0.023
